# Gene expression profiles in skeletal muscle after gene electrotransfer

**DOI:** 10.1186/1471-2199-8-56

**Published:** 2007-06-29

**Authors:** Pernille Hojman, John R Zibert, Hanne Gissel, Jens Eriksen, Julie Gehl

**Affiliations:** 1 Laboratory of the Department of Oncology, 5405, Copenhagen University Hospital Herlev, Herlev Ringvej 75, DK-2730 Herlev, Denmark; 2 Department of Dermato-Venereology KA-1502, Copenhagen University Hospital Gentofte, Niels Andersens Vej 65, DK-2900 Hellerup, Denmark; 3 Department of Physiology and Biophysics, University of Aarhus, DK-8000 Aarhus C, Denmark

## Abstract

**Background:**

Gene transfer by electroporation (DNA electrotransfer) to muscle results in high level long term transgenic expression, showing great promise for treatment of e.g. protein deficiency syndromes. However little is known about the effects of DNA electrotransfer on muscle fibres. We have therefore investigated transcriptional changes through gene expression profile analyses, morphological changes by histological analysis, and physiological changes by force generation measurements. DNA electrotransfer was obtained using a combination of a short high voltage pulse (HV, 1000 V/cm, 100 μs) followed by a long low voltage pulse (LV, 100 V/cm, 400 ms); a pulse combination optimised for efficient and safe gene transfer. Muscles were transfected with green fluorescent protein (GFP) and excised at 4 hours, 48 hours or 3 weeks after treatment.

**Results:**

Differentially expressed genes were investigated by microarray analysis, and descriptive statistics were performed to evaluate the effects of 1) electroporation, 2) DNA injection, and 3) time after treatment. The biological significance of the results was assessed by gene annotation and supervised cluster analysis.

Generally, electroporation caused down-regulation of structural proteins e.g. sarcospan and catalytic enzymes. Injection of DNA induced down-regulation of intracellular transport proteins e.g. sentrin. The effects on muscle fibres were transient as the expression profiles 3 weeks after treatment were closely related with the control muscles. Most interestingly, no changes in the expression of proteins involved in inflammatory responses or muscle regeneration was detected, indicating limited muscle damage and regeneration. Histological analysis revealed structural changes with loss of cell integrity and striation pattern in some fibres after DNA+HV+LV treatment, while HV+LV pulses alone showed preservation of cell integrity. No difference in the force generation capacity was observed in the muscles 2 weeks after DNA electrotransfer.

**Conclusion:**

The small and transient changes found in the gene expression profiles are of great importance, as this demonstrates that DNA electrotransfer is safe with minor effects on the muscle host cells. These findings are essential for introducing the DNA electrotransfer to muscle for clinical use. Indeed the HV+LV pulse combination used has been optimised to ensure highly efficient and safe DNA electrotransfer.

## Background

In vivo gene transfer to skeletal muscle is a promising strategy for treatment of muscular disorders and for systemic delivery of therapeutic proteins. Transgene expression has been reported following intramuscular injection of naked plasmid DNA [1], yet the clinical use is limited due to low efficiency and large variation. By combining intramuscular plasmid injection with local application of electric pulses a 200-fold increase in transfection efficiency with high reproducibility has been achieved in vivo [2,3]. This proves that DNA electrotransfer can be an efficient and feasible way of introducing genes into tissues. DNA electrotransfer allows high production of systemic delivered proteins e.g. erythropoietin [4,5] and cytokines [6,7] with expression detected more than a year after treatment in rodents [8,9].

The high level of gene expression in muscle after DNA electrotransfer is a consequence of plasmid distribution, membrane permeabilisation and plasmid electrophoresis [10-12]. Recently efforts have been made to optimise the electric pulses for gene transfer, resulting in a combination of a short high voltage (HV) pulse for membrane permeabilisation, followed by a long low voltage (LV) pulse for electrophoretic transport of plasmid towards the cell membrane. In skeletal muscle and skin the HV + LV pulse combination has led to increased marker gene expression [13].

DNA electrotransfer is moving rapidly towards clinical use. In fact, electroporation in combination with chemotherapy (electrochemotherapy) has been used in the clinic for several years now [14,15], showing encouraging results for anti-tumour treatment with good tolerability for the patients [16-18]. However the biological response to electroporation and DNA electrotransfer in vivo remains poorly defined. Rubenstrunk et al studied the expression of stress related genes after DNA electrotransfer, and found no significant variation between treated and non-treated muscles [19]. Furthermore, studies have shown that transient changes in force generation, muscular ion content and ATP levels were observed following DNA electrotransfer [20]. Yet no thorough description of the transcriptional changes caused by DNA electrotransfer has been reported.

In this first comprehensive microarray analysis covering the entire murine genome, we examine the transcriptional changes underlying the muscular response to DNA electrotransfer by evaluating the gene expression profiles of mice tibialis cranialis muscles 4 hrs, 48 hrs and 3 weeks after DNA electrotransfer using oligonucleotide microarrays and quantitative PCR (Q-PCR). Furthermore we performed histology and physiological tests such as force generation measurements and reflex and motor testing to support our findings.

## Results

### The effect of DNA electrotransfer on gene expression profiles

For precise description of the transcriptional changes following DNA electrotransfer, total RNA was extracted and pooled from 4 muscles excised 4 hrs, 48 hrs and 3 weeks after treatment and gene expression profile analysis was performed. Statistical analysis was employed by comparing electroporated (EP) and non-EP groups, groups either injected or not with plasmid, or groups at the different time points.

### Inferential statistics

By two-way ANOVA testing with a significance level of p = 0.001, 29 genes were found to be differentially expressed between EP and non-EP groups, 38 genes were differentially expressed in groups with or without intramuscular plasmid injection, and 145 genes were differentially expressed across time. To further filter the gene lists, unpaired t-test with 50 permutations accepting a false discovery rate (FDR) less than 5% and significance levels of p = 0.05 were performed. In the EP vs. non-EP groups, the unpaired t-test revealed that 27 of the 29 genes were significantly differentially expressed (p = 0.05) of which 7 were up-regulated and 20 down-regulated. Unpaired t-test among the groups injected with plasmid or not, revealed that 36 genes were significantly differentially expressed (p = 0.05) with 9 genes up-regulated and 27 down-regulated. Unpaired t-test among genes differing across time resulted in 130 genes with a false discovery rate of 8.5%, therefore the significance level was changed to p ≤ 0.005, resulting in 45 differentially expressed genes and a FDR of 0%.

### Descriptive statistics and unsupervised clustering

#### Electroporated versus non-electroporated muscles

**Figure 1:**
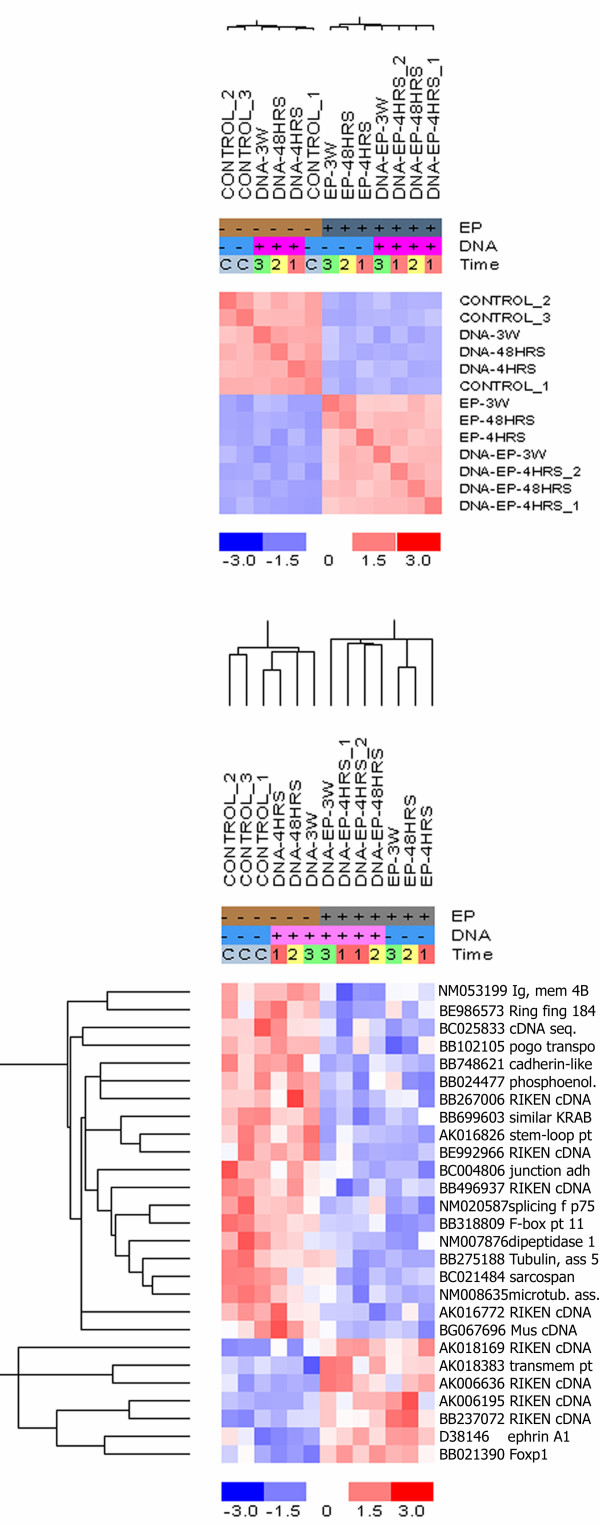
**Sample correlation matrix and hierarchical cluster analysis of gene expression profiles after electroporation**. A sample correlation matrix (upper panel) between groups, which had been electroporated or not, was built on the basis of the original two-way hierarchical unsupervised clustering. Red indicates high correlation and blue represents less correlation. The original hierarchical unsupervised clustering (lower panel) was performed with a distance measure of 1 – Pearson correlation coefficient and centroid linkage with a P-value threshold of 0.05 for significant sample clusters and significant gene clusters. The columns indicate the treatment condition and the rows indicate the individual genes, listed with their Genbank number. Increases and decreases in mRNA expression levels are represented by shades of red and blue.

In an unsupervised cluster analysis, the EP groups clustered significantly (p = 0.0006) together as depicted from the sample correlation matrix plot in Fig. 1. No correlation within the EP or non-EP clusters, as to whether the groups had received plasmid injection or at which time following treatment the muscles had been excised, was established (additional file 1).

Performing gene ontology annotation (GO) on the cluster analysis revealed that RNA encoding proteins assembling the *cytoskeleton *e.g. microtubule-associated protein 7 (NM008635), cadherin-like 26 (BB748621) and sarcospan (BC021484) were down-regulated in the electroporated groups. Yet, 3 weeks after treatment the expression of these genes in the DNA+HV+LV group had returned to control levels (Table 1).

**Table 1:** Summary of transcriptional changes following gene transfer by electroporation

Gene Ontology	EP	DNA	Time
Metabolism			
phosphoenolpuryvate carboxykinase 2	↓	0	0
dipeptidase	↓	0	0
protein phosphatase 6	0	↓	0
Cytoskeleton			
sarcospan	↓	0	0
microtubule-associated protein 7	↓	0	0
cadherin-like 26	↓	0	0
junction adhesion molecule 4	↓	0	0
Transcriptional regulation			
Ephrin	↑	0	0
Forkhead box P1	↓	0	0
F-box and leucine-rich repeat protein	↓	0	0
ring finger protein 184	↓	0	0
splicing factor (SRp75)	0	0	↑
Transducer of ERBB2	0	0	↓
eukaryotic translation initiation factor	0	0	↓
host cell factor C1	0	0	↓
CCR4-NOT transcription complex	0	0	↓
cytoplasmic polyadenylation element	0	0	0
MAPKK3	0	↓	0
polymerase II	0	↓	0
Ca^2+ ^regulation			
Reticulocalbin 2	0	↓	0
FK506 binding protein 1a	0	↓	0
Intracellular transport			
ADP-ribosylation factor-like 6 and 10	0	↓	↓
ubiquitin-like 1 (sentrin)	0	↓	0
nuclear transport factor 2	0	↓	0
Membrane transport			
Chloride channel Ka	0	0	↑
Kruppel-like factor 15	0	0	↓
K^+ ^voltage gated channel, Shab related	0	0	↓
Solute carrier family 6	0	0	↓
ATP-binding cassette (ABC1)	0	0	↓

Furthermore expression of genes involved in the *metabolism *e.g. phosphoenolpuryvate carboxykinase (BB024477) and dipeptidase (NM007876) were also down-regulated following electroporation, indicating that the general catabolism might be depressed. Ephrin (D38146), which is involved in neural development and cell-cell interaction among vascular endothelial cells were found to be up-regulated in all EP groups.

#### Plasmid vs. no plasmid injection

**Figure 2:**
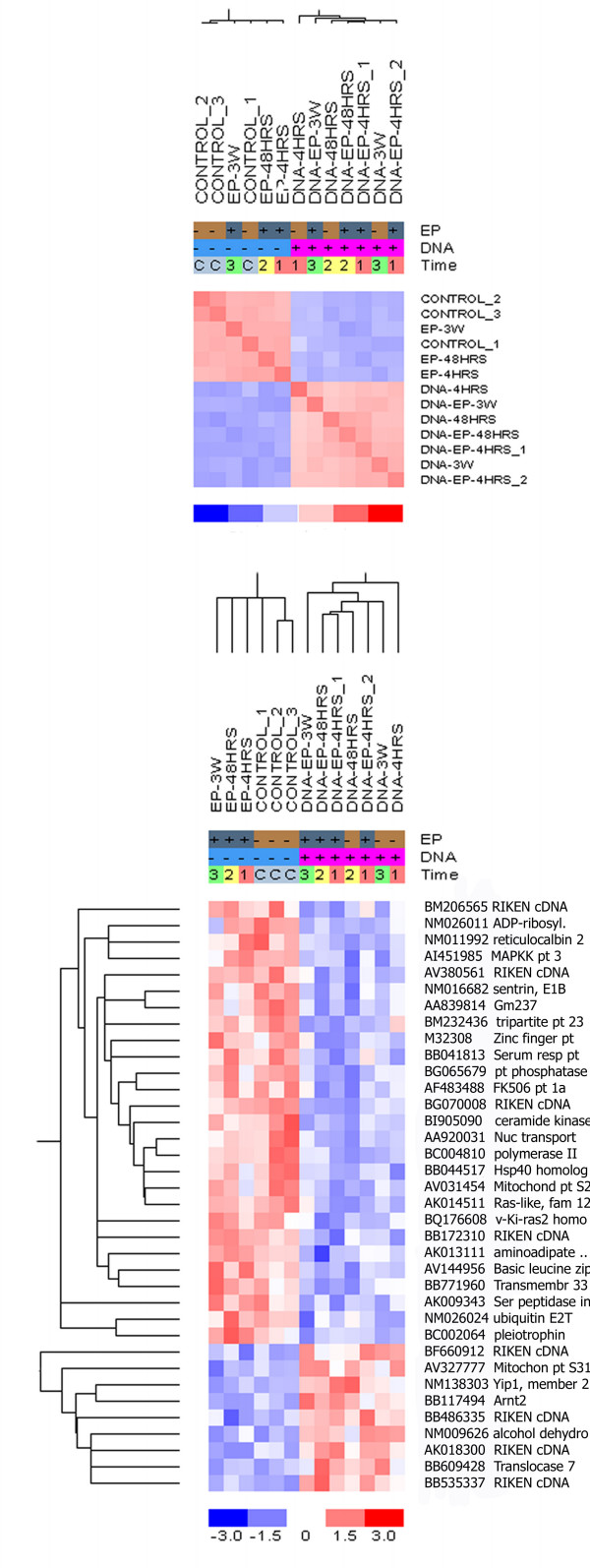
**Sample correlation matrix and hierarchical cluster analysis of gene expression profiles after DNA injection**. A sample correlation matrix (upper panel) between DNA injected or non-injected groups was built on the original two-way hierarchical unsupervised clustering. Red indicates high correlation and blue represents little correlation. In the original hierarchical unsupervised clustering (lower panel) the columns indicate the treatment condition and the rows indicate the individual genes, listed with their Genbank number. Increases and decreases in mRNA expression levels are represented by shades of red and blue.

The plasmid injected groups clustered significantly together (p = 0.0006) in the sample correlation matrix analysis with clear separation from the non-injected groups (Fig. 2). Interestingly the LDA plot revealed that within the plasmid injected groups, two apparent sub-clusters of EP and non-EP groups were found. Likewise among the non-injected groups, the EP groups formed a distinct sub-cluster (additional file 2).

GO annotation showed that the expression of genes involved in *intracellular trafficking *e.g. sentrin (ubiquitin-like 1, NM016682), nuclear transport factor 2 (AA920031) and ADP-ribosylation factor-like 10C (NM026011) were down-regulated in the plasmid injected groups. In addition polymerase II (BC004810) was also down-regulated in plasmid injected groups, indicating the *transcription *also was depressed.

Furthermore the ER Ca^2+ ^binding protein reticulocalbin (NM011992) and Ca^2+ ^release modulator FK506 binding protein (AF483488) were both down-regulated following plasmid injection, which might be important for controlling the *Ca*^2+ ^*homeostasis*. Indeed we have previously shown that DNA injection especially in combination with electroporation affects the intracellular level of Ca^2+ ^(manuscript in preparation).

#### Time

**Figure 3:**
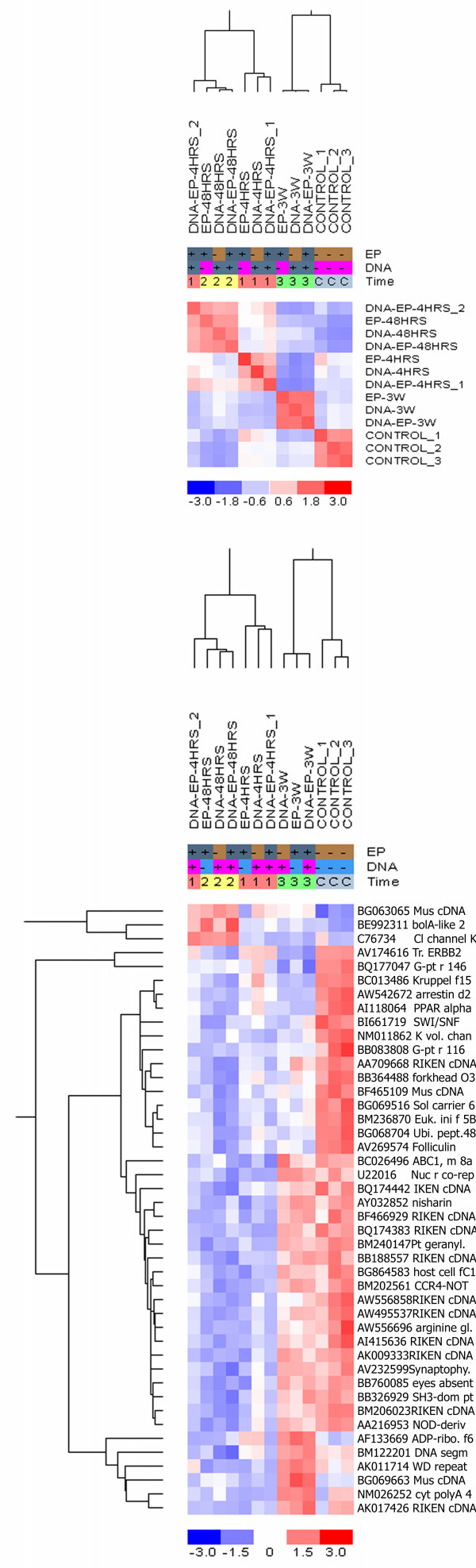
**Sample correlation matrix and hierarchical cluster analysis of gene expression profiles at different time points**. A sample correlation matrix (upper panel) was built from groups evaluated at different time points. Red indicates high correlation and blue represents little correlation. In the original hierarchical unsupervised clustering (lower panel) the columns indicate the treatment condition with the designation 1 = 4 hours, 2 = 48 hours, 3 = 3 weeks and C = control. The rows indicate the individual genes, listed with their Genbank number. Increases and decreases in mRNA expression levels are represented by shades of red and blue.

Groups within each of the 4 time points clustered significantly together (Fig. 3) regardless of the treatment. The LDA plot revealed that groups from 4 and 48 hrs clustered closely together, while the control and 3 weeks groups formed individual clusters (additional file 3).

At 4 hrs the chloride channel K_a _(C76734) is up-regulated in all groups compared to all other time point. At this initial time point after treatment and membrane permeabilisation, the *ion homeostasis *across the membrane might still be disturbed, stimulating the expression of the Cl-channel. At 48 hrs and in the EGT-treated group at 4 hrs, the transducer of ERBB2 (AV174616), was up-regulated. Up-regulation of this inhibitor of proliferation might indicate disturbances in the muscles, which limit the proliferation potential transiently.

Compared to the control groups, the Cl^- ^channel activator Kruppel-like factor 15 (BC013486) and the K^+ ^voltage-gated channel (NM011862) were down-regulated at 4 hrs, 48 hrs and 3 weeks, indicating the *ion homeostasis *might be disturbed longer than the initial phase. Similarly the solute carrier family 6 protein (bg069516), which transports creatine into the muscle cells and is essential for energy consumption, was also down-regulated at all time points compared to the control groups.

At 4 and 48 hrs the *transcription activator *host cell factor C1 (bg864583), the regulator of polymerase II CCR4-not transcription complex (BM202561) and the polyadenylation element binding protein (NM026252) were down-regulated compared to the control and 3 weeks groups; indicating that the protein synthesis apparatus might be negatively affected within the first 48 hrs after treatment.

### Q-PCR validation of differentially expressed genes

The gene expression levels were validated by Q-PCR for 11 selected genes (Table 2). These measurements confirmed the level of mRNA determined by microarray analysis. For example transducer of ERBB2 (AV174616) was in the array analysis found to be up-regulated at 48 hours after all treatments, indeed Q-PCR also showed that the transducer of ERBB2 was up-regulated at 48 hours e.g. in the case of DNA+HV+LV treatment by 6-fold. Similarly the expression of the solute carrier family 6 (BG069516), responsible for creatine transport, was verified by Q-PCR to be down-regulated at 4 and 48 hours.

**Table 2:** Validation of differential expressed genes after DNA electrotransfer

		Inj. alone		DNA alone			HV + LV			DNA + HV + LV		
		4 H	48 H	4 H	48 H	3 W	4 H	48 H	3 W	4 H	48 H	3 W

Trans ERBB2	1,00	4,52	9,35	4,66	6,10	5,61	4,17	5,25	3,34	1,02	6,30	-1,83
FK506 binding pt.	1,00	0,68	-0,44	2,73	0,56	0,94	0,75	0,63	0,52	1,17	0,53	0,86
Solute carrier family	1,00	-0,79	-1,07	0,11	-0,34	0,25	-0,60	-0,66	0,05	0,59	-0,56	0,83
Reticulocalbin 2	1,00	0,89	0,24	3,19	0,55	0,91	0,95	0,82	0,62	1,19	0,53	1,00
Ceramide kinase	1,00	0,41	-0,85	2,97	0,73	1,00	0,59	0,53	0,45	1,02	0,81	1,27
Phosphoenol pur.	1,00	-1,99	-4,06	-0,42	-3,39	0,36	-2,29	-2,58	-1,59	-1,14	-3,39	0,02
Nuc. transport factor	1,00	1,16	0,72	3,21	0,57	0,93	1,05	1,00	0,68	1,24	0,52	1,03
Polymerase II	1,00	1,31	0,55	3,85	0,61	1,10	1,25	1,15	0,85	1,39	0,68	1,15
Sarcospan	1,00	1,14	-0,55	4,93	-0,17	0,88	0,65	0,40	0,46	3,26	0,55	1,65
Ubiquitin-like	1,00	0,00	-0,08	2,79	0,06	0,89	-0,09	-0,19	0,34	0,92	0,00	0,82
Ca-channel	1,00	0,21	0,00	3,39	0,21	1,14	0,48	-0,13	0,69	1,24	0,12	0,79

Eleven genes were selected for Q-PCR validation of their expression level. The genes were normalised to β-microglobulin and down and up-regulation in expression compared to the control muscles were determined.

### Histological changes in tibialis cranialis muscles after DNA electrotransfer

**Figure 4:**
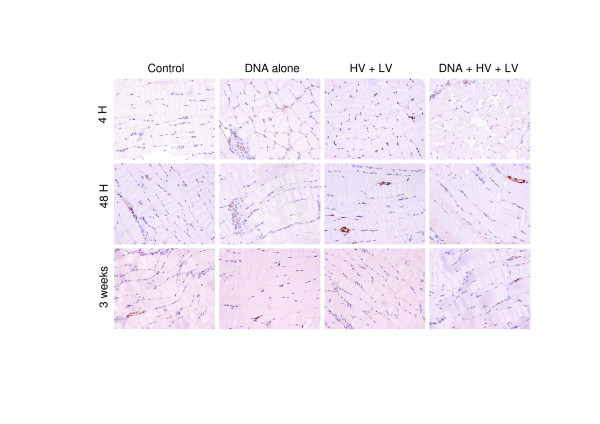
**Histological changes after DNA electrotransfer to TC muscles**. Muscles were excised 4 hours, 48 hours and 3 weeks after DNA electrotransfer, and fixed and stained with hematoxylin and peroxidase-conjugated CD4 antibodies. Representative pictures from 4 slides from the different conditions are depicted.

Preparations of muscles slides were stained with HE and examined for morphological changes like integrity of fibres, central nuclei and fainting striation pattern. Furthermore, CD4 and CD8 staining were performed on parallel slides to verify infiltration of mononuclear cells. At 4 hrs, no histological changes were observed for the control, DNA injected and electroporated muscles (Fig. 4). Contrarily after DNA+HV+LV treatment, loss of fibre structure and fainting striation pattern was observed in 15% of the muscle. After 48 hrs, no changes were observed in the control and DNA injected muscles, while the electroporated muscles exhibited fainting striation pattern in a few muscle fibres. In the DNA+HV+LV treated muscles, structural changes with loss of cell integrity and striation pattern were observed in 15% of the fibres, in addition limited intra-fiber infiltration of mononuclear cells was also observed. Three weeks after treatment, the DNA+HV+LV treated muscles had around 5% of the fibres containing central nuclei, indicating regenerating myocytes. In the electroporated muscles single cells with central nuclei were also detectable, while the control and DNA injected muscles showed no signs of histological changes.

### No loss in muscle performance after DNA electrotransfer

**Figure 5:**
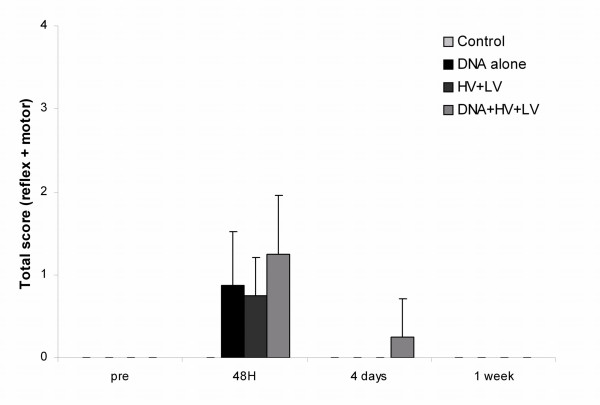
**Reflex and motor function in mice after DNA electrotransfer *in vivo***. Mice were tested for reflex and motor function according to a system, where 0 = normal function, 1 = function affected and 2 = function gravely affected. The total score (reflex + motor) is depicted for the following time points pre-treatment, 48 hours, 4 days and 1 week and (n = 12).

The reflex and motor function of mice were tested by assessing the running and seizing patterns. Thus, mice were allowed to run on the top of the cage, their tails were gently manually lifted, and it was observed whether they could grasp the bars with normal force. Similarly, spontaneous motoric movement was observed in the cage. Fig. 5 depicts the average score, short duration films of the test results can be found in additional file 4, 5, 6, 7, 8, 9, 10, 11, 12, 13, 14, 15, 16, 17, 18, 19. At 4 hours the mice had not fully awoken from the anaesthesia and could therefore not be tested. At 48 hours no effects were observed in the control and DNA injected mice, while slight impairments were observed in a couple of the electroporated and DNA+HV+LV treated mice. From 5 days after treatment no functional impairments in the seizing and running patterns were observed in any of the mice.

**Figure 6:**
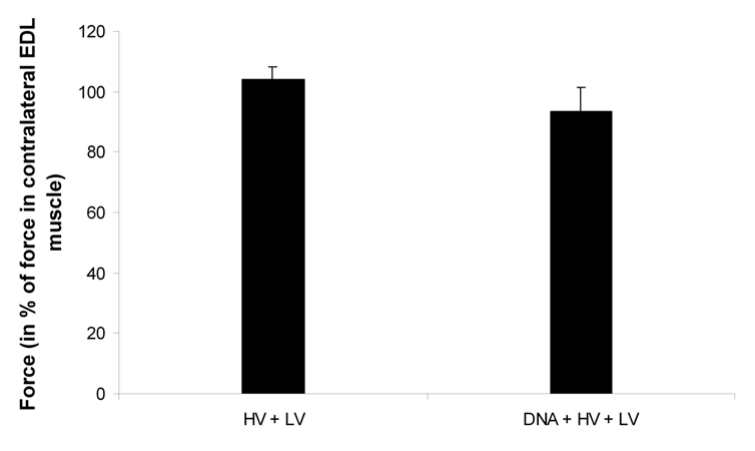
**Force recovery in rat EDL muscle after DNA electrotransfer *in vivo***. The right EDL muscle of rats was electrotransferred with 5 μg pGFP-S65T (DNA+HV+LV) or only subjected to electric pulses (HV+LV). The left EDL muscle served as untreated control. Two weeks after DNA electrotransfer the animals were sacrificed and the EDL muscles were excised and placed in a force displacement transducer. Force was measured using 90 Hz pulse trains with duration of 0.5 s. Force of the treated muscle was recorded and related to force in the contralateral control muscle. Mean values with bars denoting SD are given, n = 4 muscle sets. There were no significant difference between the treated and the control muscles.

The effects of DNA electrotransfer on the force generating capacity were tested in rat EDL muscles 2 weeks after treatment and depicted in Fig. 6. Rat EDL muscles were chosen for this measurements as mice TA cannot be used in our set-up. The force generating capacity in the DNA electrotransferred and electroporated muscles did not differ significantly from the control muscles.

## Discussion

DNA electrotransfer to skeletal muscle is highly efficient and holds great promise for future treatment of monogenetic disorders, protein deficiency syndromes, cancers, etc. For electric pulsing of muscle in vivo trains of long low field strength pulses have typically been employed [2,21]. However studies have shown that the electric pulse-mediated gene transfer might consist of two components; a component, which permeabilises the membrane, and an electrophoretic component, which can facilitate the transport of plasmid DNA across the membrane [10,12]. Using combination pulses, a short high voltage permeabilising pulse followed by a long low voltage electrophoretic pulse, less intense pulsing parameters are needed for obtaining the same expression levels, which reduces the discomfort from the pulsing regiment. Indeed in this study we confirm these observations as little changes in the gene expression profiles are observed. Moreover we find less histological changes than previously described after DNA electrotransfer with trains of long, low field strength pulses [22,23]. In addition we find no reductions in muscle performance 2 weeks after treatment, indicating that no major muscle regeneration is required after DNA electrotransfer.

### Array analysis, clustering and time aspect

Due to the expenses of the study, the replica number was low in this study, however this was accommodated by designing our statistical analysis as a multiple factor analysis. During the inferential statistics we performed an ANOVA test, which gives an efficient and comprehensive evaluation despite the low replicate number in this set-up [24]. Moreover, permutation test and unpaired T-tests were performed using stringent level of significance to correctly identify genes that were differentially expressed. Furthermore extensive descriptive statistics were performed, which supported the findings of differentially expressed genes. Unsupervised cluster analyses showed clear separations between EP and non-EP muscles and DNA injected or non-injected muscles, indicating that the differentially transcribed genes in these clusters were highly characteristic for the given treatment. Indeed some of the changes we observed were in line with previous reported changes following DNA electrotransfer to cells. Not surprisingly the gene expression profiles at 4 and 48 hrs were relatively comparable, while the gene expression profiles after 3 weeks were more similar to the control muscles. This indicates that the observed changes after DNA electrotransfer are transient, followed by a return to the naïve state of the muscle. The most striking point about the expression profiles is that no genes involved in stress, immune responses, cell death or muscle regeneration were found to be differentially expressed, demonstrating that EGT is non-toxic. Indeed guided supervised cluster analysis looking at cell homeostasis, ion homeostasis and immune response ontologies showed no significant changes in the gene expression profiles.

### DNA electrotransfer-induced transcriptional changes

Based on previous in vitro studies certain effects of electroporation and plasmid injection would have been expected. Electroporation of cells is known to cause cytoskeleton rearrangements and electro-conformational changes of ion channels, while DNA injection is associated in cytotoxic effects on the cells.

### Changes in cytoskeleton-associated proteins

During the application of an electric field across cells, electro-compressive forces exert pressure on the membrane leading to elongation of cells. During this elongation phase the pressure on the membrane enlarges, resulting in the development of membrane destabilisations, which allow for entry of plasmids [25,26]. Formation of microvilli and blebs associated with deformation of the cytoskeleton has been observed immediately after pulsing [27,28]. In fact, the same phenomenon is observed in cyclic stretch-induced gene transfer, where cytoskeleton rearrangements are involved in mediating the gene transfer [29]. In this study we found down-regulation of cytoskeleton proteins after electro-pulsing, corresponding to the reported deformations of the cytoskeleton after pulsing. Interestingly, studies have shown that muscle contractile function returns to normal within 4 hours after EGT with the HV+LV pulse combination (manuscript in preparation), indicating that the cytoskeleton rearrangements only have a minor effect on the muscle function.

### Electro-conformational changes of ion channels

During electro-pulsing, voltage-dependent ion channels are particularly sensitive to the external electric field due to their intrinsic voltage sensors. High intensity electric fields cause structural changes, also known as electro-conformational changes, which especially affect the moveable charge particles in the channel [30,31]. Chen et al have shown that the movement of charge particles in the delayed rectifier K+ channels was reduced after applying an external transmembrane potential of 400 mV [31,32]. This applied membrane potential is considerably larger than the 150 – 200 mV required for lipid bilayer breakdown. At lower than 400 mV field intensities no current changes demonstrating conformational changes were observed [31]. In our study we found down-regulation of the K^+ ^voltage-gated channel. Although our applied electric field does not reach amplitude of 400 mV at the membrane level, weaker field strengths might be sufficient to induce electro-conformational changes of ion channels in muscle fibres, as these cells are larger. This suggests that electro-conformational changes might play a role in the regulation of voltage-gated channels after DNA electrotransfer in vivo.

### Effects of plasmid injection

Intramuscular injection of plasmid can be associated with toxicity in the target tissue [33,34]. Hartikka et al (2001) observed that muscle damage after DNA electrotransfer with super-coiled endotoxin-free plasmid was DNA dependent. When introducing 50 μg of plasmid into the quadriceps muscle, they observed lesions with necrotic myofibres and infiltration of inflammatory cells. After injection of vehicle and electric pulses delivery no such lesions were observed. We have used a low concentration of plasmid, which might explain why we observed little infiltration at our histological evaluation and no effects on the transcriptional level.

### Intracellular plasmid DNA transport

Unexpectedly we found that proteins involved in intracellular transport were down-regulated following DNA injection. After entry into muscle fibres plasmid DNA must be transported to the nuclei for transcription, but as the cytoplasmic latticework is quite condense, it is unlikely that this transport occur by simple diffusion [35]. In HeLa and COS cells it has been shown that plasmid DNA has a limited half-life (1 – 2 hours) in the cytosol due to degradation by cytoplasmic nucleases [36,37], meaning that efficient transport to the nucleus is required. In fact studies have shown that the cytoplasmic movement of DNA towards the nucleus in cells is facilitated by the microtubular network [38]. The down-regulated intracellular transport proteins are not directly involved in these transport pathways, but they belong to a group of proteins, which might be a limiting factor in efficient gene transfer. This could therefore be of importance for the transfection efficacy.

## Conclusion

DNA electrotransfer by HV+LV pulses demonstrates small and transient adverse effects on the muscle. This is highly important for introduction in the clinical setting, where numerous applications of this technology are envisaged. Examples of interesting pre-clinical experiments with gene transfer to muscle tissue include correction of anaemia [39-42] or β-thalassemia [43,44] by electrotransfer of EPO. Furthermore DNA electrotransfer can also be used to target the muscle itself, either for research purposes or for correction of muscle anomalies e.g. muscular dystrophies. In this regard, it is important that the gene transfer procedure itself imposes minimal effects on the target tissue.

In conclusion DNA electrotransfer using the HV+LV pulse combination is safe with only small changes in the expression of cytoskeletal and intracellular transport proteins, while most importantly no detrimental changes in muscle performance or gene expression profiles of proteins involved in cell death, inflammation or muscle regeneration was verifiable both shortly and long after treatment. Histology did, however, revealed some cells with morphological changes after 4 and 48 hrs and few cells containing central nuclei after 3 weeks.

## Methods

### Animals

Six to eight weeks old female C57Black/C mice (average weight 22 g, Taconic, Denmark) or 4 – 5 weeks old male or female Wistar rats (own breed, University of Aarhus) were kept under pathogen-free conditions at 22°C in a 14/10 hrs light/dark cycle with food and water ad libitum. All animal experiments were conducted in accordance with the recommendations of the European Convention for the Protection of Vertebrate Animals used for Experimentation and after permission from the Danish Animal Welfare Committee.

### Plasmid DNA and in vivo DNA electrotransfer

The plasmid phGFP-S65T, encoding the green fluorescent protein (GFP), was obtained from Clontech (Palo Alto, CA, USA). DNA preparations were performed using Qiafilter Plasmid Maxiprep kits (Qiagen, Germany), and the concentration and quality of the plasmid preparations were controlled by spectrophotometry, accepting a 260/280 ratio of 1.8–1.9 and by gel electrophoresis.

Five micrograms of plasmid was dissolved in 20 μl PBS (Phosphate-buffered saline without MgCl_2_, the Hospital Pharmacy at Herlev, Denmark), and the plasmid solution was injected intramuscularly along the fibres into the tibialis cranialis muscle of anaesthetised C57Bl/6 mice using an insulin syringe. For rats, 5 μg/10 μl PBS were injected into the EDL muscle. Plate electrodes with 4-mm gap were fitted around the hind legs and an electric field was applied using the Cliniporator™ (IGEA, Italy). The muscle was pulsed with a combination of a high voltage pulse (100 μsec, 1000 V/cm) followed by a long low voltage pulse (400 ms, 100 V/cm) with 1 s lag between the pulses.

### RNA extraction and gene expression analysis

Four hrs, 48 hrs and 3 weeks after treatment the mice were euthanized and the muscles were removed and placed in 1 ml solution D (guandinium thiocyanat, sodium citrate, sarcosyl and mercaptoethanol) on ice. Muscle extracts were prepared by homogenising the muscles with a rotor-stator homogeniser (S8N – 5G, IKA-werke, Germany), and RNA were extracted using the Chomczynski and Sacchi method [45] and digested with DNase (Promega, Ramcon, Denmark). The extracted RNA amount ranged from 5 – 33 μg with a mean concentration of 14.3 +/- 7.9 μg.

Due to low yield of total RNA, mRNA were amplified yielding antisense RNA (aRNA) using the MessageAMP II aRNA kit (Ambion, Europe). Briefly, total RNA were reverse transcribed into first strand cDNA using a T7 Oligo(dT) primer and M-MLV reverse transcriptase and incubated at 42°C for 2 hrs. Second strand cDNA was synthesized using DNA polymerase and purified on cDNA filter cartridge according to the manufacturers protocol. An in vitro transcription (IVT) reaction was set up to generate multiple copies of aRNA from the double stranded cDNA through incubation at 37°C for 14 hrs.

Affymetrix microarray analyses were preformed at the Affymetrix core facility at the Microarray Center at National University Hospital. In short 5 μg aRNA was used to synthesize double stranded cDNA using Superscript^® ^Choice System (Invitrogen, Denmark) with an oligo(dT) primer containing a T7 RNA polymerase promoter (GenSet, Evry, France). The cDNA was used as template for an IVT reaction to generate biotin-labelled antisense cRNA (BioArray™ High Yield RNA Transcript Labelling Kit; Enzo Diagnostics, Farmingdale, NY, USA). After fragmentation at 94°C for 35 min in fragmentation buffer (40 mM Tris, 30 mM MgOAc, 10 mM KOAc), the labelled cRNA were hybridised for 16 hrs to Affymetrix MOE 430 2.0 arrays (Affymetrix, Santa Clara, CA, USA). The arrays were washed and stained with phycoerythrin conjugated streptavidin using the Affymetrix Fluidics Station^® ^400, and the arrays were scanned in the Affymetrix GeneArray^® ^scanner to generate fluorescent images, as described in the Affymetrix GeneChip protocol.

### Microarray data analysis and statistics

The cel image files (Affymetrix) were imported, pre-processed and analysed in DNA-Chip Analyser 2006. The array files were normalised using the non-linear invariant set normalisation method, choosing array (DNA-EP-48 HRS) as baseline. Normalisation curves were visually inspected and none of the arrays were critical. In order to interpret the probe signal, model-based gene expression indexes (MBEI) [46] gene expression modelling was calculated using the PM/MM (perfect match/miss match) method. During the analysis array outliers were detected. None of the arrays exceeded 1.4% of array outliers and the probe presence call was between 36.6% and 50.3%. The experiment conditions and replicate number were *electroporation *(n = 13 with n_EP+ _= 7 and n_EP- _= 6), *DNA injection *(n = 13 with n_DNA+ _= 7 and n_DNA- _= 6) and *time interval *(n = 13 whereas n_ctrl _= 3, n_time 4 hrs _= 4, n_time 48 hrs _= 3 and n_time 3 weeks _= 3)

Raw data from the experiment are available at the gene Expression Omnibus database (GEO) in a MIAME compliant format. They are submitted under the serial code GSE6686, and under the following sample codes GSM154212, GSM154215, GSM154216, GSM154218, GSM154222, GSM154226, GSM154230, GSM154233, GSM154234, GSM154235, GSM154236, GSM154237, GSM154238.

*Inferential statistics *were adapted to the data by two-way ANOVA testing with a significance level of p ≤ 0.001, which have been described to give high power in multiple factor experiments [24]. To further filter the gene lists, unpaired t-test with 50 permutations accepting a false discovery rate (FDR) less than 5% and significant level of p = 0.05 were performed. An unpaired t-test among genes differing across time resulted in 130 genes with a false discovery rate of 8.5%, therefore the significance level was changed to p ≤ 0.005, with a result of 45 differentially expressed genes and a FDR of 0%.

*Descriptive statistics *were applied to the significant data to obtain biologically meaningful patterns.

Unsupervised two-way hierarchical clustering was performed with a distance measure of 1 – Pearson correlation coefficient and centroid linkage with a P-value threshold of 0.05 for significant sample clusters and significant gene clusters [46].

A sample correlation matrix (SCM) was built on the basis of the original hierarchical unsupervised clustering, providing valuable information of how efficiently the clusters correlated. Supervised analyses were performed by applying specific issue algorithms of interest (downloaded from NetAffx™ Analysis Center). The issues of interest were cell homeostasis, ion homeostasis, ion channel activity and immune responses. To obtain significant data the data was filtered by an unpaired t-test with a significance level of p ≤ 0.05 and variation across samples between 0.50 < Standard deviation/mean < 1000.00. No FDR criterion was applied because we wanted to see a possible existing pattern. Furthermore to find a linear combination of explanatory variables and coefficient estimates within the three groups of interest (EP, Plasmid, time) the difference between the distributions of linear discriminate scores (LDA-plot) for each group was maximized.

Finally the supervised analysis tool gene ontology (GO) was applied to the data. Data from the two-way hierarchical clustering was uploaded to two software programs EASE and NetAffx™ Analysis Center, which have the abilities to calculate the GO similarity. The comparison criterions within the groups were set to the highest similarity within the genes of same gene category.

### Quantitative PCR

Two-step real-time reverse transcription polymerase chain reaction (RT-PCR) was performed to validate samples of differentially regulated genes in the electrotransferred muscles as determined by interferential statistic analysis. The aRNA prepared for the array hybridisation was reverse transcribed using random p(dN)_6 _primers (Roche, Germany) and AMV reverse transcriptase (Roche, Germany). Eleven selected genes were amplified from the cDNA using the Brilliant SYBR Green Q-PCR kit (Stratagene, AH Diagnostics, Denmark). The primers were designed using Primer3 and PCR and detection was performed by Q-PCR (MX3000P, Stratagene, AH Diagnostics, Denmark). The RNA levels were normalised to β-microglobulin and down and up-regulation in expression compared to the control muscles were determined.

### Evaluation of macroscopic and histological changes and GFP expression in tibialis cranialis muscles

Respectively 4 hrs, 48 hrs and 3 weeks after treatment the mice were euthanized and whole tibialis cranialis muscles were carefully isolated and evaluated for macroscopic changes. The isolated muscles were either evaluated for GFP expression by fluorescence stereo microscopy (Nikon SMZ1500, Japan) in blinded and randomised order or fixed in 1 ml formalin buffer and imbedded in paraffin blocks following standard procedures. Sections 3 – 5 μm thick were stained with hematoxylin and eosin (Mayer-Sour) or with alkaline phosphatase-conjugated CD4 antibodies followed by counterstaining with Mayer's hematoxylin.

### Test of function

The treated animals were tested for their ability to seize a grid by placing the animal on the grid and lifting the hind part by the tail. Animals with normal muscle function were given the score 0, while animals with affected function were rewarded 1 and those, who did not seize the grid at all were scored 2. For running the animals were placed alone in an empty cage. Normal running were given the score 0, while animals with a limp were given 1 and animals that did not support on the leg were rewarded 2. A combined score from the two tests were then calculated.

### Measurement of force

Force was measured as previously described in detail by Clausen & Everts [47]. In brief, isolated EDL muscles were mounted vertically with their tendons intact on a force displacement transducer (Grass FT03, W. Warwick, RI, USA) for isometric contractions in thermostatically controlled (30°C) chambers containing standard Krebs-Ringer bicarbonate buffer (pH 7.3) with (in mM): 122.1 NaCl, 25.1 NaHCO_3_, 2.8 KCl, 1.2 KH_2_PO_4_, 1.2 MgSO_4_, 1.3 CaCl_2_, and 5 D-glucose and gassed continuously with a mixture of 95% O_2 _and 5% CO_2_. Direct electrical stimulation was delivered via platinum electrodes on either side of the mid-portion of the muscle. Muscle length was adjusted to optimal length during repeated stimulation with single pulses. Finally force was checked using short tetanic contractions induced by supramaximal 1 ms pulses of 10 V at 90 Hz for 0.5 sec.

## Abbreviations

EGT – DNA electrotransfer, EP – electroporation, GO – gene ontology, HV – high voltage, LV – low voltage.

## Authors' contributions

PHM carried out the experimental work and drafted the manuscript. JRZ performed the microarray analysis and statistics and helped revise the manuscript. HG performed the force generation measurements. JE participated in the design of the study. JG participated in the design of the study, interpretation of results and drafting the manuscript. All authors have read and approved the final manuscript.

## Supplementary Material

Additional file 1 **Linear discriminate analysis based on electroporated or non-electroporated groups**. Red represents the electroporated groups and blue represents the non-electroporated groups. Click here for file

Additional file 2 **Linear discriminate analysis based on DNA injected or non-injected groups**. Red represents the DNA-injected groups and blue represents the non-injected groups. Click here for file

Additional file 3 **Linear discriminate analysis based on groups evaluated at different time points**. Blue indicate groups evaluated after 4 hours, red represents groups evaluated after 48 hours, green indicates groups evaluated after 3 weeks and light blue represents control groups. Click here for file

Additional file 4 **pre group 1 run**. Representative film of running pattern in mice in group 1 (control) before treatment. Click here for file

Additional file 5 **pre group 1 seize**. Representative film of seizing pattern in mice in group 1 (control) before treatment. Click here for file

Additional file 6 **pre group 2 run**. Representative film of running pattern in mice in group 2 (DNA alone) before treatment. Click here for file

Additional file 7 **pre group 2 seize**. Representative film of seizing pattern in mice in group 2 (DNA alone) before treatment. Click here for file

Additional file 8 **pre group 3 run**. Representative film of running pattern in mice in group 3 (HV + LV) before treatment. Click here for file

Additional file 9 **pre group 3 seize**. Representative film of seizing pattern in mice in group 3 (HV + LV) before treatment. Click here for file

Additional file 10 **pre group 4 run**. Representative film of running pattern in mice in group 4 (DNA + HV + LV) before gene electrotransfer. Click here for file

Additional file 11 **pre group 4 seize**. Representative film of seizing pattern in mice in group 4 (DNA + HV + LV) before gene electrotransfer. Click here for file

Additional file 12 **48 H group 1 run**. Representative film of running pattern in mice in group 1 (control) 48 hours after treatment. Click here for file

Additional file 13 **48 H group 1 seizing**. Representative film of seizing pattern in mice in group 1 (control) 48 hours after treatment. Click here for file

Additional file 14 **48 H group 2 run**. Representative film of running pattern in mice in group 2 (DNA alone) 48 hours after treatment. Click here for file

Additional file 15 **48 H group 2 seize**. Representative film of seizing pattern in mice in group 2 (DNA alone) 48 hours after treatment. Click here for file

Additional file 16 **48 H group 3 run**. Representative film of running pattern in mice in group 3 (HV + LV) 48 hours after treatment. Click here for file

Additional file 17 **48 H group 3 seize**. Representative film of seizing pattern in mice in group 3 (HV + LV) 48 hours after treatment. Click here for file

Additional file 18 **48 H group 4 run**. Representative film of running pattern in mice in group 4 (DNA + HV + LV) 48 hours after gene electrotransfer. Click here for file

Additional file 19 **48 H group 4 seize**. Representative film of seizing pattern in mice in group 4 (DNA + HV + LV) 48 hours after gene electrotransfer. Click here for file
