# Supplementary figures and images for: Gene expression profiles in skeletal muscle after gene electrotransfer
Source: BMC Mol Biol. 2007 Jun 29;8:56. doi: 10.1186/1471-2199-8-56 (PMC1925113; doi:10.1186/1471-2199-8-56)

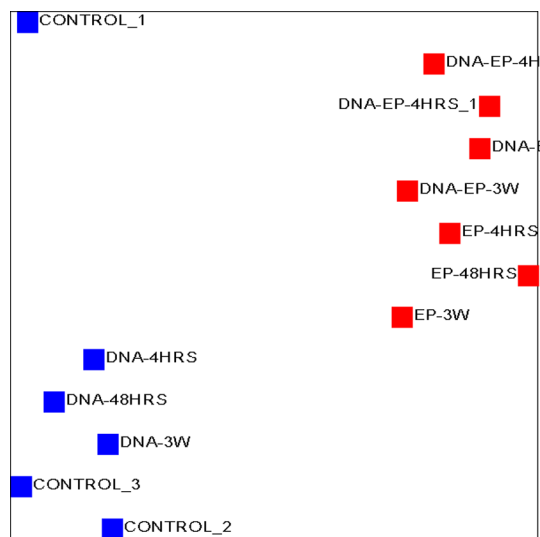

Supplement: Additional file 1 — Linear discriminate analysis based on electroporated or non-electroporated groups. Red represents the electroporated groups and blue represents the non-electroporated groups. [file 1471-2199-8-56-S1.pdf]

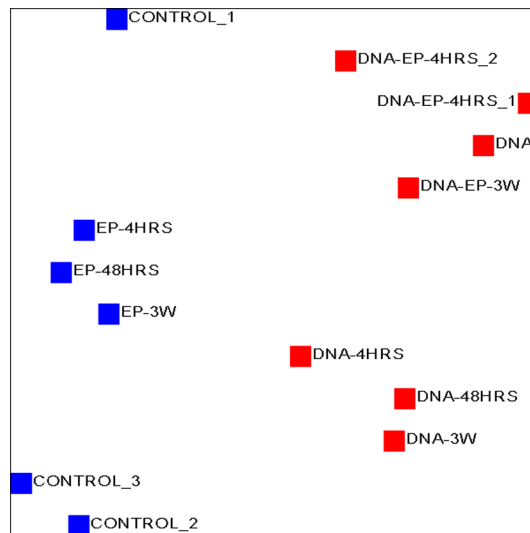

Supplement: Additional file 2 — Linear discriminate analysis based on DNA injected or non-injected groups. Red represents the DNA-injected groups and blue represents the non-injected groups. [file 1471-2199-8-56-S2.pdf]

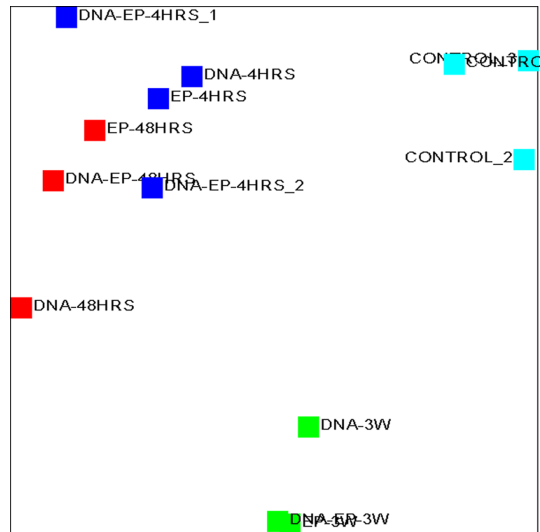

Supplement: Additional file 3 — Linear discriminate analysis based on groups evaluated at different time points. Blue indicate groups evaluated after 4 hours, red represents groups evaluated after 48 hours, green indicates groups evaluated after 3 weeks and light blue represents control groups. [file 1471-2199-8-56-S3.pdf]
